# Factors associated with renal Doppler resistive index in critically ill patients: a prospective cohort study

**DOI:** 10.1186/s13613-019-0500-4

**Published:** 2019-01-31

**Authors:** Raphael A. G. Oliveira, Pedro V. Mendes, Marcelo Park, Leandro U. Taniguchi

**Affiliations:** 1 Surgical Emergencies and Trauma ICU, Hospital das Clinicas HCFMUSP, Faculdade de Medicina, Universidade de Sao Paulo, Av Enéas de Carvalho Aguiar 255, São Paulo, SP Postal Code: 05403-000 Brazil; 2 Emergency Medicine Discipline, Hospital das Clinicas HCFMUSP, Faculdade de Medicina, Universidade de Sao Paulo, Av Enéas de Carvalho Aguiar 255 Sala 5023, São Paulo, SP Postal Code: 05403-000 Brazil; 3 Hospital Sirio Libanes, Rua Daher Cutait 69, São Paulo, SP Postal Code: 01308-060 Brazil

**Keywords:** Acute kidney injury, Renal resistive index, Intensive care unit, Chloride, Critical care, Doppler ultrasonography

## Abstract

**Background:**

The renal Doppler resistive index (renal RI) is a noninvasive tool that has been used to assess renal perfusion in the intensive care unit (ICU) setting. However, many parameters have been described as influential on the values of renal RI. Therefore, we proposed this study to evaluate the variables that could impact renal RI in critically ill patients.

**Methods:**

A prospective observational study was performed in a 14-bed medical–surgical adult ICU. All consecutive patients admitted to the ICU during the study period were evaluated for eligibility. Renal RI was performed daily until the third day after ICU admission, death, or renal replacement therapy (RRT) requirement. Clinical and blood test data were collected throughout this period. Acute kidney injury (AKI) reversibility was categorized as transient (normalization of renal function within 3 days of AKI onset) or persistent (non-resolution of AKI within 3 days of onset or need for RRT). A linear mixed model was applied to evaluate the factors that could influence renal RI.

**Results:**

Eighty-three consecutive patients were included. Of these, 65% were male and 50.6% were medical admissions. Mean SAPS 3 was 47 ± 16. Renal RI was significantly different between no-AKI (0.64 ± 0.06), transient AKI (0.64 ± 0.07), and persistent AKI groups (0.70 ± 0.08, *p* < 0.01). Variables associated with renal RI variations were mean arterial pressure, lactate, age, and persistent AKI (*p* < 0.05). No association between serum chloride and renal RI was observed (*p* = 0.868).

**Conclusions:**

Mean arterial pressure, lactate, age, and type of AKI might influence renal RI in critically ill patients.

## Background

Acute kidney injury (AKI) is a common condition in critically ill patients with high morbidity and mortality rates, particularly in cases of persistent AKI [1]. The renal Doppler resistive index (renal RI) has recently been suggested as a bedside tool in critically ill patients. It is a rapid and noninvasive technique that allows evaluation of renal hemodynamics through the analysis of flow velocities through the renal arterioles obtained by pulsed Doppler ultrasonography [2–5]. In recent years, renal RI has been used with a wide variety of renal conditions for diagnostic and prognostic evaluation, such as evaluation of renal graft rejection after transplantation [6, 7], assessment of the progression of chronic renal disease [8], and evaluation of renal artery stenosis in patients with systemic arterial hypertension [9, 10]. In the intensive care unit (ICU) setting, a recent meta-analysis showed that renal RI might be an accurate tool to assess the reversibility of AKI in critically ill patients [2].

However, it has been acknowledged that the name of the method is unfortunate, since many systemic and local factors other than vascular resistance are probably more relevant to the values given by renal RI (e.g., arterial compliance, mean arterial pressure, and systemic vascular disease markers) [11–13]. Nevertheless, the factors that might influence renal RI in critically ill patients are not yet fully understood [5, 14] since previous studies were all in other settings. Notably, some data suggested a relationship between serum chloride and renal vascular resistance [15]. Thus, the goal of our study was to evaluate the factors that could impact renal RI in critically ill patients.

## Materials and methods

### Settings and patients

A prospective observational study was performed in a 14-bed medical–surgical intensive care unit (ICU), from November 2013 to October 2014, approved by local ethics committee (Hospital das Clínicas, University of São Paulo, Brazil-Protocol Number 335.619). As the data were collected during standard routine care performed in the ICU, the same committee waived the need for informed written consent. Since previous data suggested an association between serum chloride and renal vascular resistance [15], we evaluated the association between these two variables in a pilot observation with the first 15 patients included. This analysis was planned before data collection, and it was necessary due to the lack of data regarding the association between chloride and renal RI. We observed a positive correlation between these two parameters (Spearman correlation, *ρ* = 0.348, *p* < 0.05). Thus, with *α* = 0.05 and a *β* = 0.10, we estimated a sample size of 83 patients [16].

Inclusion criteria were all patients admitted to the ICU with an expected length of stay longer than 72 h. Exclusion criteria were age under 18 years, pregnancy, known artery renal stenosis, chronic kidney disease defined by a glomerular filtration rate of less than 30 mL/min/1.73 m^2^, cirrhosis with hepatorenal syndrome, use of renin–angiotensin–aldosterone antagonists, absence of a vesical indwelling catheter during the study period, and AKI on renal replacement therapy (RRT) or with an expectation of RRT within 24 h.

### Study protocol

Each patient included was studied within the first 24 h after ICU admission. The same operator performed renal RI daily until the third day after ICU admission, death, or RRT requirement, whichever occurred first. A fully trained investigator who performed the measurements was not in charge of the patients, and the physicians in charge were unaware of the results of the renal RI. RI was performed only after hemodynamic stabilization defined as the mean arterial pressure greater than 65 mmHg for more than 1 h without any fluid loading or any change in the rate of catecholamine infusion. The ventilator settings and sedative infusion rates (if applicable) were unchanged for at least 1 h before renal RI.

An ultrasound machine (GE Healthcare^®^ LOGIQ P5, Wisconsin, USA) with a 4 MHz curved-array transducer was used. Renal RI was obtained from a posterolateral approach from the right kidney in all but three patients. B mode allowed kidney localization and detection of signs of chronic renal disease. An interlobar or arcuate artery was identified and then selected. The Doppler spectrum was considered optimal when at least three similar consecutive waveforms were visualized. The peak systolic velocity (*V*_max_) and the minimal diastolic velocity (*V*_min_) were determined by pulse wave Doppler. RI was calculated as (*V*_max_ − *V*_min_)/*V*_max_. At least, three recordings were obtained from the selected arteries, and the mean of three RI was used to the analysis [3].

Patient demographics, SAPS 3 score [17], SOFA score [18], comorbidities, daily urine output and fluid balance, use of vasopressors, use of loop diuretics, use of mechanical ventilation, and renal replacement therapy requirement during the first 3 days of the ICU stay were recorded. Both ICU and hospital mortality were also recorded. Blood samples were collected routinely, once daily, just after renal RI measurements.

### AKI diagnosis and reversibility

AKI was evaluated at admission and daily during the ICU stay; it was defined according to KDIGO criteria [19], which proposed AKI as any of the following: increase in serum creatinine (sCr) greater or equal to 0.3 mg/dL within 48 h; or increase in sCr to greater or equal than 1.5 times baseline that was known or presumed to have occurred within the prior 7 days; or urine output less than 0.5 mL/kg/h for 6 h. Baseline serum creatinine was defined as the lowest value in the previous 3 months before ICU admission. In the absence of known baseline sCr, the nadir of sCr after renal recovery was used. In the absence of renal recovery, baseline sCr was estimated by using the Modification of Diet in Renal Disease (MDRD) formula.

AKI’s reversibility was categorized as transient or persistent. Transient AKI was defined as a 50% decrease in sCr or normalization of urine output within 3 days. Persistent AKI was defined as persistent elevated sCr, oliguria for at least 72 h or need of RRT [20].

### Data analysis

Continuous parametric and nonparametric variables were presented as the mean (standard deviation) and median (25th; 75th percentiles) and were compared using the *t* test and Mann–Whitney test, respectively. Categorical variables were expressed as absolute (*n*) and relative (%) frequency and were compared by the Chi-square test with Yates correction. Comparison between three groups was made using Kruskal–Wallis and ANOVA for nonparametric and parametric variables, respectively. Correlations tests were performed using the Spearman correlation coefficient.

A linear mixed model was applied to evaluate the association between renal RI values and the variables of interest and to account for repeated measures. Renal RI was assessed as a dependent variable, and hemodynamic data, SAPS 3, presence of sepsis, use of vasoactive drugs, age, AKI category (absent, transient or persistent), and laboratory values were evaluated as fixed covariates.

All analyses and graphs were generated using R project 3.0.2 (www.r-project.org) and SPSS Statistics 19 (Chicago, Illinois, USA). A *p* value of less than 0.05 was considered statistically significant in all cases.

## Results

**Fig. 1:**
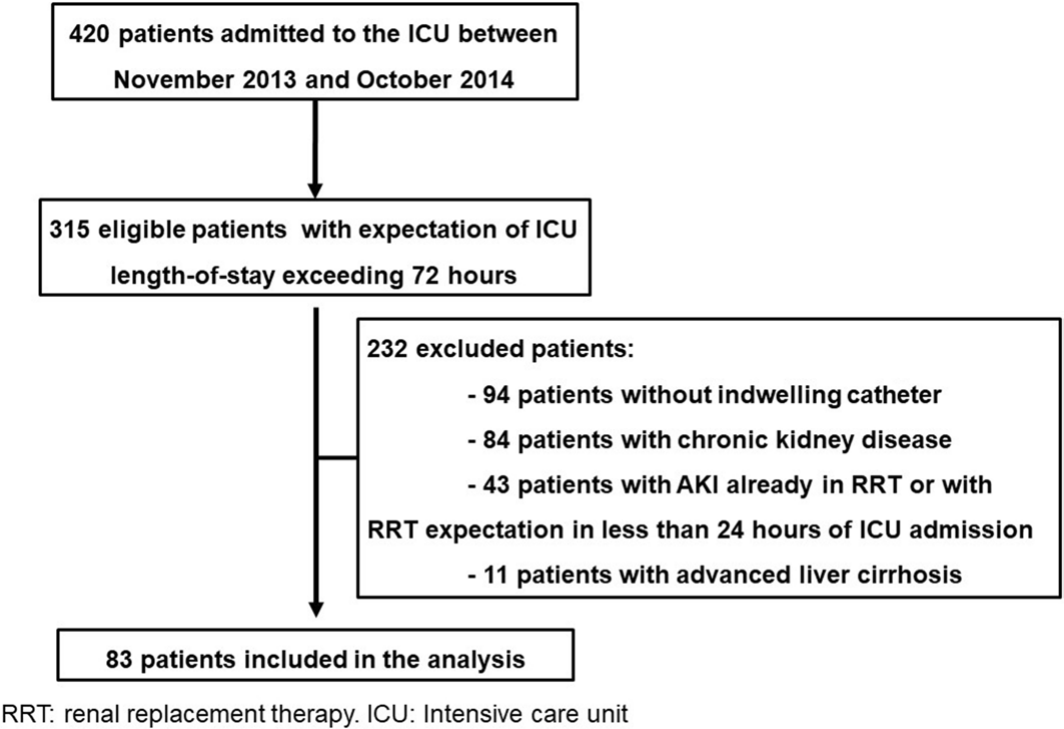
Flowchart of patients eligible and included in this study

Eighty-three consecutive patients were included in the study. The flowchart with the total of eligible patients during the period and the exclusion criteria are listed in Fig. 1. General characteristics of patients upon ICU admission according to AKI category (no AKI, transient AKI, and persistent AKI) developed during the observation period are described in Table 1. No differences were observed between the three groups when variables such as age, gender, need for mechanical ventilation, and need for vasoactive drugs were analyzed. There were also no statistically significant differences among the rates of comorbidities presented in the various groups. SAPS 3 was significantly higher in the group of patients who developed persistent AKI than in the group of patients who developed transient AKI and the group who did not develop AKI during the observation period. The main cause of ICU admission was multiple traumas followed by neurological syndromes and sepsis, with respiratory infections being the most frequent cause (52%). The ICU and hospital mortality rates were significantly higher in the group of patients who developed persistent AKI than in the other groups (*p* = 0.03).

**Table 1:** Baseline characteristics of patients and main outcomes according to acute kidney injury categorization

Characteristics	No AKI (*n*: 21)	Transient AKI (*n*: 25)	Persistent AKI (*n*: 37)	Overall (*n*: 83)
Male gender, *n* (%)	10 (47)	20 (80)	24 (65)	54 (65)
Medical admission, *n* (%)	8 (38)	11 (44)	23 (62)	42 (51)
Age, years	50 (38; 59)	43 (22; 64)	54 (32; 69)	51 (31; 64)
SAPS 3	41 ± 15^a^	45 ± 14^a^	53 ± 12	47 ± 16
Mechanical ventilation, *n* (%)	12 (57)	14 (56)	16 (43)	42 (51)
Vasopressor therapy, *n* (%)	5 (24)	11 (44)	18 (49)	34 (41)
Main diagnosis, *n* (%)				
Multiple trauma	3 (14)	12 (48)	7 (19)	22 (26)
Neurological syndromes	7 (33)	5 (20)	8 (22)	20 (24)
Sepsis	3 (14)	2 (8)	13 (35)	18 (22)
Acute abdomen	2 (9)	4 (16)	3 (8)	9 (11)
Associated diseases, *n* (%)				
Arterial hypertension	9 (42)	8 (32)	17 (46)	34 (41)
Diabetes mellitus	4 (19)	2 (8)	8 (22)	14 (17)
Solid neoplasms	2 (9)	3 (12)	5 (13)	10 (12)
Chronic heart failure	1 (5)	1 (4)	8 (22)	10 (9)
Coronary arterial disease	3 (14)	0 (0)	4 (11)	6 (8)
RRT^b^, *n* (%)	0	0	8 (22)	8 (10)
ICU mortality^b^, *n* (%)	4 (19)	5 (20)	14 (38)	23 (27)
Hospital mortality^b^, *n* (%)	4 (19)	5 (20)	17 (46)	26 (31)

*RRT* renal replacement therapy, *ICU* intensive care unit, *AKI* acute kidney injury

^a^*p* < 0.05 compared to persistent AKI

^*b*^*p* < 0.05, using Chi-square with Yates correction

Sixty-two patients (74%) developed acute renal injury during the study period; 53 patients (64%) already had the diagnostic criteria of AKI on admission to the ICU and the other nine patients (10%) developed AKI during the first 24 h after admission. A total of 58% of patients who already had AKI on admission developed persistent AKI, and 42% had transitory AKI. Sixty-six percent of patients who developed AKI in the first 24 h after admission to the ICU showed changes consistent with persistent AKI. Only 13% of patients who developed AKI during the study period needed RRT.

The systemic, hemodynamic, and laboratory variables collected during the study period, according to the AKI category, are described in Table 2. Hemodynamic parameters such as heart rate, mean arterial pressure, serum lactate levels, and need for vasoactive drugs were not different among the groups. Twenty-four-hour fluid balances were similar among the groups on the first 2 days of observation, but with negative mean values on the third day in the group of patients without AKI (*p* < 0.05 between groups). There were no differences in serum chloride values among the groups during the period.

**Table 2:** Hemodynamic and laboratorial data according to acute kidney injury category

	No AKI	Transient AKI	Persistent AKI	*p*
Mean arterial pressure (mmHg)				
Day 1	86 ± 16	87 ± 13	84 ± 15	0.55
Day 2	89 ± 16	88 ± 11	86 ± 18	0.85
Day 3	86 ± 12	90 ± 17	84 ± 12	0.72
Pulse pressure (mmHg)				
Day 1	35 ± 6	35 ± 6	34 ± 6	0.78
Day 2	36 ± 7	34 ± 5	35 ± 7	0.84
Day 3	34 ± 5	37 ± 8	35 ± 5	0.69
Norepinephrine use (%)				
Day 1	19	32	38	0.33
Day 2	18	30	44	0.27
Day 3	8	10	27	0.26
Heart rate (bpm)				
Day 1	83 ± 18	93 ± 20	92 ± 22	0.18
Day 2	81 ± 16	86 ± 15	91 ± 19	0.16
Day 3	80 ± 13	85 ± 14	95 ± 20	0.05
Lactate (mmo/L)				
Day 1	1.88 (1.66; 2.55)	1.88 (1.44; 2.77)	2.33 (1.77; 3.11)	0.18
Day 2	1.83 (1.44; 2.19)	1.72 (1.36; 2.11)	2.33 (1.36; 2.11)	0.16
Day 3	1.77 (1.5; 2.0)	1.55 (1.11; 2.33)	2.0 (1.66; 2.33)	0.48
Serum chloride (mEq/L)				
Day 1	107 ± 7.8	105 ± 5	106 ± 6	0.71
Day 2	106 ± 7	108 ± 5	107 ± 8	0.79
Day 3	106 ± 6	106 ± 6	108 ± 9	0.69
Fluid balance—24 h (mL)				
Day 1	273 (− 429; 675)	454 (− 520; 849)	311 (70; 981)	0.58
Day 2	89 (− 272; 358)	217 (− 168; 1950)	839 (98; 1600)	0.07
Day 3	− 467 (− 621; 473)^a,b^	961 (636; 1624)	531 (34; 1275)	0.01
Renal RI				
Day 1	0.65 ± 0.077^b^	0.64 ± 0.07^b^	0.71 ± 0.08	< 0.01
Day 2	0.63 ± 0.07^b^	0.64 ± 0.06^b^	0.71 ± 0.09	< 0.01
Day 3	0.64 ± 0.05	0.64 ± 0.07	0.69 ± 0.08	0.12

*RI* resistive index, *AKI* acute kidney injury

^a^*p* < 0.05 compared to transient AKI

^b^*p* < 0.05 compared to persistent AKI

**Fig. 2:**
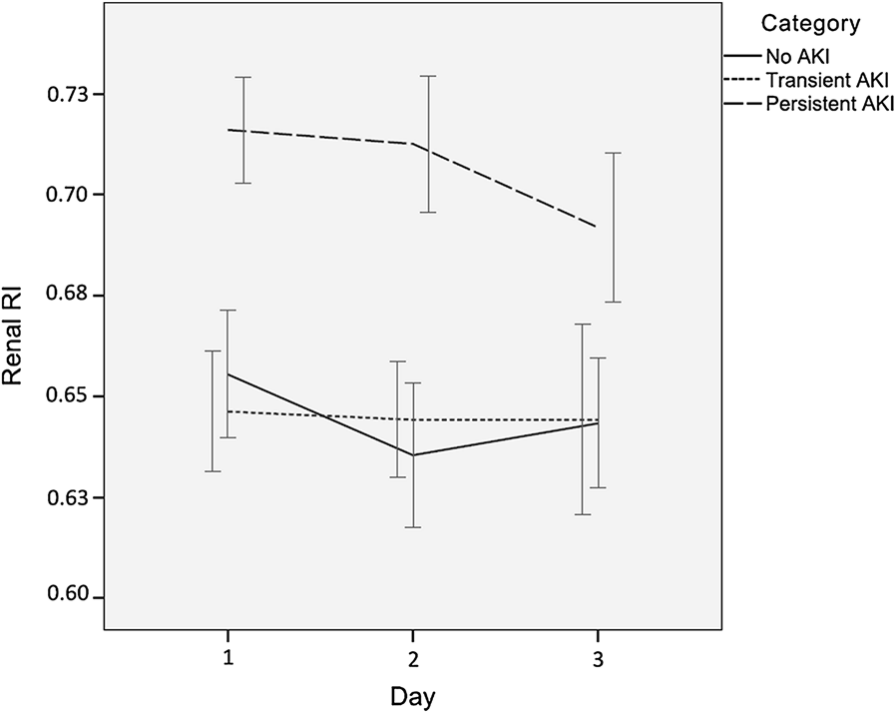
Mean renal RI values according to acute kidney injury category. *AKI* acute kidney injury, *RI* renal resistive index. Error bars represent ± 1 standard error of the mean

Renal RI behavior according to AKI category during the study is shown in Fig. 2. Consistently higher values of renal RI were observed in patients with persistent AKI than in patients with transient AKI or patients without AKI on days 1 and 2 (*p* < 0.05).

The linear mixed model results to assess the influence of potential variables on renal RI are described in Table 3. The only variables that demonstrated an association with renal RI were mean arterial pressure, lactate, presence of persistent acute renal injury, and age. Serum lactate and age were positively associated with renal RI, whereas mean arterial pressure displayed a negative association in the model. There was no association between serum chloride and renal RI (*p* = 0.868).

**Table 3:** Variables associated with renal RI affording linear mixed model results

Parameter	Coefficient	Standard error	95% confidence interval		*p*
			Lower bound	Upper bound	
Age (years)	0.0018	0.0003	0.0011	0.0026	< 0.01
AKI persistent	0.0474	0.1821	0.0837	0.0110	0.01
Lactate (mmol/L)	0.0108	0.0034	0.0040	0.0176	< 0.01
Mean arterial pressure (mmHg)	− 0.0007	0.0002	− 0.0013	− 0.0001	0.01

Model included sepsis, SAPS 3, age, serum chloride, vasopressor therapy, mean arterial pressure, heart rate, lactate, and AKI according to its reversibility (fixed effects)

*AKI* acute kidney injury

## Discussion

In this study, renal RI values during the ICU stay were significantly higher in the group of patients with persistent AKI than in the other groups. Variables associated with renal RI were age, mean arterial pressure, serum lactate levels, and presence of persistent acute renal injury, but serum chloride levels were not associated with renal RI. The interactions between factors that influence renal RI are multifaceted, and understanding the associations among these variables is essential to correctly interpret the technique.

Renal RI values showed very specific behavior during the study, with changes according to the presence and evolution of AKI, and were consistently higher in the group of patients with persistent AKI than in the other groups of patients without AKI or with transient AKI. These latter groups exhibited similar renal RI behavior. It is believed that the transient AKI behavior is due to only reversible and non-structural functional changes [3, 21, 22]. Therefore, the fact that we did not observe a difference between renal RI values in patients without AKI and patients with transient AKI suggests this pathophysiological foundation.

Thus, it is important to understand the factors that may influence renal RI in critically ill patients. We evaluated the influence of variables known to be associated with renal RI and those with evident pathophysiological background to influence the method. The variables such as sepsis, SAPS 3, age, serum chloride, use of vasoactive drugs, mean arterial pressure, heart rate, lactate, and presence of acute kidney injury according to its reversibility were included in the model. Only age, lactate, mean arterial pressure, and presence of persistent acute renal injury were associated with renal RI.

The association between acute kidney injury and renal RI suggests a pathophysiological reasoning, reflecting the renal structural alterations in the tubule-interstitial and vascular compartments and consequent increases in renal impedance values [14, 23]. On the other hand, the positive association between age and renal RI, observed in the model, potentially reflects vascular changes due to aging, with loss of large vessel compliance and increased aortic impedance [6, 24–27].

The negative association between mean arterial blood pressure and renal RI was intriguing. It might reflect the role of systemic blood pressure in renal vascular resistance. Increased mean blood pressure may lead to a reduction in renal vascular resistance due to flow-induced renal vasodilation or due to increasing the number of perfused renal vessels, leading to lower renal RI values [28, 29]. Although the mechanism of this association has not been completely elucidated, it has been described since 1988 [30] and observed by others [28, 29].

To the best of our knowledge, the positive association of renal RI and lactate was first described in this study. After analyzing lactate as an isolated severity marker, the study found no differences between values of lactate according to AKI category during the observation period, although higher mean lactate values were observed in the group of patients with persistent AKI. The positive association observed between renal RI and lactate could indicate that high renal RI values may be associated with greater clinical severity. These data are corroborated by a recent description of an independent association of renal RI with ICU mortality in patients with high renal RI values measured at the time of AKI diagnosis [31].

No association was observed between serum chloride and renal RI in the final model. Although serum chloride had a significant physiological relevance in the determination of renal vascular resistance through the depolarization of smooth muscle cells of the renal arterioles [32, 33], chloride-dependent vasoconstriction of the renal arterioles would only be a part of one of the determinants of renal RI. Recently, randomized clinical trials reported no association between infusions of chloride-rich solutions and clinical outcomes such as acute perioperative renal injury in patients undergoing heart surgery [34] and AKI in critically ill patients [35]. Our data were consistent with these latest findings, to the detriment of the several observations in physiological studies regarding the positive association between serum chloride and renal RI [5].

Overall, our findings suggest an association between illness severity and renal RI. It could be speculated that through many different pathophysiological pathways, those patients with higher severity (those with low blood pressure who are older with more pronounced arterial stiffness, hyperlactatemia, and established AKI) have higher renal impedance that translates into higher renal RI values. This might explain previous observations correlating renal RI with worse outcomes [2].

## Limitations

The study has a number of limitations that must be considered in order to assess the relevance of the findings. First, it was a single-center observational study with a heterogeneous population. The findings may not have sufficient external validation. Second, as we used quite rigorous exclusion criteria that were necessary for the proper control of pre-analytical variations for a robust analysis of renal RI, some selection bias may have occurred in the inclusion of patients, possibly compromising the reliability of our findings in the general population of critically ill patients. Third, we, like previous investigators [3, 20, 28], did not measure intra-abdominal pressure in all patients; intra-abdominal pressure is known to affect renal RI [36]. Finally, only one operator performed all renal RI measurements. Although the intensivist performing the measurements was fully trained in the technique, we did not perform a blinded control of a random sample to access reliability.

## Conclusions

In critically ill patients, age, mean arterial pressure, serum lactate levels, and presence of persistent acute renal injury were identified as influencing renal RI, whereas serum chloride was not found to have an influence. More studies are needed to confirm our findings.

## Abbreviations

Renal RI: renal Doppler resistive index
ICU: intensive care unit
RRT: renal replacement therapy
AKI: acute kidney injury
SAPS 3: Simplified Acute Physiology Score 3
SOFA: Sequential Organ Failure Assessment
MDRD: Modification of Diet in Renal Disease
